# The Association of Children’s Blood Lead Levels and Prevalence of Stunting in Tin Mining Area in Indonesia

**DOI:** 10.5334/aogh.4119

**Published:** 2023-08-28

**Authors:** Rismarini Zarmawi, Budi Haryanto

**Affiliations:** 1 Department of Environmental Health, Faculty of Public Health, Universitas of Indonesia, ID; 2 Research Center for Climate Change, Universitas Indonesia, ID

**Keywords:** children, blood lead levels, stunting, tin mining, Indonesia

## Abstract

**Background:**

Metal mining and smelting activities are regarded as major sources of heavy metals such as lead, mercury, arsenic and cadmium in the environment and in humans living at the surrounding area. Among others, lead can enter and accumulate in the human body and be very influential in children’s growth and development.

**Objective:**

This study aims to assess the association between children’s blood lead levels and stunting in a mining area in Indonesia.

**Methods:**

A cross-sectional design was implemented by involving 193 children living in surrounding tin mining in Bangka Island, Indonesia. Venous blood was drawn and blood lead level was measured by Inductively Coupled Plasma Mass Spectrometry (ICP-MS). Stunting status was measured by anthropometry standing height and converted to sexand age-specific Z-scores based on World Health Organization (WHO) growth reference. Children’s dietary intake was assessed using 24-hour dietary recall method. Statistics of chi-square test and multiple logistic regression were performed for the analyses.

**Findings:**

The geometric mean of Blood Lead Levels (BLLs) was 5.5 µg/dl (± 2.6 µg/dl; 95% CI: 5.1–5.9). The interquartile range of BLLs and height for age Z-score (HAZ) were 3.0 μg/dl and –1.5, respectively. The data revealed that 23.3% of children were stunted (HAZ < –2). The multiple logistic regression models suggest that elevated BLLs were an independent predictor of the stunting. The odds stunted blood lead concentration was elevated about 10times higher [adjusted odd ratio (AOR) = 9.75 (95% Confidence interval (CI): 3.1–30.7); p < 0.001] in comparison to the odds of normal BLLs.

The BLLs of children at ages two to nine years were found associated with stunting after controlling of the mother›s education, residence and the intake of energy, protein, zinc, vitamin A, calcium and phosphorus.

**Conclusion:**

The study suggested that living in surrounding tin mining was dangerous for children›s health and their development.

## Introduction

Lead remains a potential toxin for children even at low concentrations [1] and still as a severe health concern since there is no acceptable blood lead level [2]. Children’s blood lead levels (BLLs) and lead exposure in the environment are closely connected [3,4]. Most currently, children’s BLLs were utilized to determine their level of lead exposure [5]. In previous studies, several sociodemographic variables were significantly associated with children’s blood lead concentration and with anthropometric measurements of the children [6]. Several studies have been found to address how undernutrition could increase lead absorption [7]. Furthermore, poor nutrition and other socioeconomic-related factors may contribute to the simultaneous existence of stunting and elevated BLLs [8]. Several studies have shown an association between increased BLLs and decreased height [9] but did not clearly address the incidence of stunting. Government and commercial enterprises are the owners of the tin mining business permits for the Bangka Belitung Islands Province. Most of the West Bangka district’s mainland tin output is concentrated in Muntok District, where 11 furnaces at PT. Timah Tbk’s smelting plant with a 42,000-ton processing capacity are in operation. West Bangka Regency is in danger of environmental lead contamination due to its long history as a hub for tin exploration and manufacturing. Children nearby the mining area may be at risk of lead toxicity due to the operation of large-scale tin mining in West Bangka Regency. The production of tin ore leaves behind tailings, which can be a source of lead exposure, as can the refining of tin in hightemperature kilns used at smelter sites. The sole district in the Bangka Belitung Islands Province, selected by the Indonesian government as one of the 100 priority cities or districts for stunting intervention, is West Bangka Regency. Based on the results of the 2013 National Social and Economic Survey (SUSENAS), the stunting in West Bangka Regency was reported to be 33.2% while the 2013 Basic Health Study (Riskesdas) found 39.1% stunting. Four of the 10 priority villages for stunting intervention in 2017—Ibul, Peradong, Simpang Tiga and Tugang—still have stunting prevalence rates above 20%. This study intends to examine the relationships between sociodemographic, dietary and blood lead levels and the prevalence of stunting.

## Methods

In July 2020, an observational study with a cross-sectional method was implemented for measuring the prevalence and factors associated with stunting in Muntok and Simpang Teritip, which are two sub-districts in the West Bangka. Purposively, 228 mothers and 228 children aged between two to nine years were selected and measured for their anthropometry, maternal, socio-demographic and children’s nutrients intake. However, blood lead samples were available only from 193 children. We obtained parental or caregiver informed consent prior to blood sampling. Venous blood was drawn from the cubital vein into an evacuated, heparinized 3 ml venoject tube. Blood lead levels were measured by ICP-MS. It is a multielement technique that uses an inductively coupled plasma (a very high temperature ionized gas composed of electrons and positively charged ions) source to atomize the sample and subsequently ionize the atoms of interest [10]. The ions were extracted from the plasma and passed through a mass spectrometer where they were separated and measured based on their mass-to-charge ratio. The efficiency of the inductively coupled plasma in producing ions from the atoms of interest in the aerosolized sample, coupled with the high selectivity of the quadrupole (which filters the ions), the high amplification of ionic signals striking the detector and the low background noise of the detector, provides very low instrument detection limits (parts per trillion to low parts per billion) for most elements. ICP-MS method detection limits for direct analysis of lead in blood are approximately 0.1 µg/dl [11]. Concentrations of unknown samples were determined against the standard curve. All tests were carried out at the Jakarta Medical Laboratory Center. Children’s dietary intake was assessed using 24-hour dietary recall methods conducted by trained interviewers. A combination of household measures (cup, spoon, rice ladle) and food models were used to estimate the weight of reported food consumed. Mothers or caregivers were asked about the meals that were consumed by children in the previous 24-hour period. All foods and drinks consumed by children in the last 24 hours were reported, including details of portion size, brands and cooking processes. Finally, additional probing for confirmation was performed to ensure the accuracy and completeness of foods reported. The results of the 24–hour recall were converted into nutrient intakes using the Nutrisurvey, *y*a software for transforming foods/dishes consumption to nutrients. Nutritional intake is grouped based on the percentage of compliance with the Nutrition Adequacy Rate (RDA) for Indonesian children based on age (with a range of 1–3, 4–6 and 7–9 years). Assessment of stunting was done following the World Health Organization (WHO) Multicentre Growth Standard. Measured standing height of all participants were converted to sex and age-specific Z-scores based on WHO growth reference. Children’s height for age Z-score (HAZ) were calculated using the WHO Anthro and AnthroPlus. A z-score of less than –2 standard deviations was considered as stunting [13]. Children’s characteristics including sex, birth weight, residential, history of chronic diarrheal and environmental tobacco smoke exposure, and other demographic characteristics like education and occupation of parents, were measured using a questionnaire that was answered by the mothers. Height-for-age was derived based on the WHO standard, summarised and presented as means and standard deviations, and used to construct categorical variables. To identify children’s blood lead levels, we used a blood lead reference value of 5 micrograms per decilitre (µg/dL) [14].

Statistics descriptive analysis—bivariate using the chi-square test, and logistic regression to determine predictors of stunting—were performed. The association between stunting and independent variables were first analysed using bivariate logistic regression model. Bivariate logistic regression analysis is using two random variable responses, which is Y_1_ = 0 and Y_2_ = 1. If both of Y_1_ and Y_2_ has correlation, thus there will be four levels of the binary response pair, then it can be labelled as (1,1) for Y_1_ = 1 and Y_2_ = 1, (1,0) for Y_1_ = 1 and Y_2_ = 0, (0,1) for Y_1_ = 0 and Y_2_ = 1, and (0,0) for Y_1_ = 0 and Y_2_ = 0. Covariates having p-value <0.25 were retained and entered to the multivariable logistic regression analysis. A p-value < 0.05 was considered as a cut-off point for an independent variable to be significantly associated with the outcome [12]. Multiple logistic regression was used to observe any independent association between the outcome variable (stunting) and elevated BLL while regressing mother’s education, mother’s age at birth, childhood diarrhoea history, child’s environment, residence and nutrient intake as potential confounders.

This study was approved by the Research and Community Engagement Ethical Committee of Faculty of Public Health Universitas Indonesia (NKB-261/UN2.F10.D11/PPM.00.02/2020). Informed written consent was taken from the parents of the children. All consent givers were briefed and assured about non-disclosure of the information and use of the analysed data findings for scientific purposes.

## Results

A total of 193 children(122 girls and 71 boys) donated blood samples. The mean (± standard deviation) BLL was 5.5 µg/dl (± 2.6 µg/dl; 95% CI: 5.1–5.9), with the minimum and maximum BLL were 1.0 and 20.0 µg/dl. The interquartile range BLL and HAZ were 3.0 μg/dl and –1.5, respectively. Of all the subjects whose data was analysed, 57.5% of the children had elevated BLL (> 5 µg/dL). More than half were deficient of energy (61.7%), vitamin A (68.9%), vitamin C (85%), vitamin D (67.9%) and calcium (91.2%). About one-fourth of the children (23.3%) were stunted (HAZ < –2). The mean value of four nutrient intake was not sufficient for the RDA. The mean energy, calcium, vitamin A and phosphorus intake were 77.5 kcal, 33.4 mg, 81.6 mg, and 140.9 mg, respectively. However, the mean intake of protein, zinc, vitamin D and magnesium exceeded the RDA. Additionally, more than half (51.3%) of the mothers of all children did not attend senior secondary school. The distribution of the overall children’s characteristics and nutrient intake found the BLLs average to be 5.5 μg/dL and the children’s age about seven-years-old (Table 1).

**Table 1:** Summary distribution of children’s characteristics and nutrient intake (N = 193).


VARIABLE	MEAN (±SD)	95% CI

Blood Lead Levels (µg/dL)	5.5 (± 2.6)	5.1, 5.9

HAZ	–1.0 (±1.2)	–1.2, –0.9

Child’s age (years)	6.9 (±1.4)	6.7, 7.1

Child’s birth weight	3,090.7 (± 469.2)	3024.5, 3156.9

Mother’s age at birth (years)	29.4 (± 26.2)	25.7, 33.1

Energy intake (%RDA)	77.5 (± 28.5)	73.5, 81.5

Protein intake(%RDA)	48.6 (± 21.3)	45.6, 51.6

Zinc intake (%RDA)	100.5 (± 69.0)	90.8, 110.3

Calcium intake (%RDA)	33.4 (± 40.3)	27.7, 39.1

Vitamin A intake (%RDA)	81.6 (± 127.1)	63.6, 99.5

Vitamin C intake (%RDA)	39.3 (± 52.9)	31.9, 46.8

Vitamin D intake (%RDA)	60.1 (± 56.6)	52.2, 68.1

Phosphorus intake (%RDA)	140.9 (± 66.6)	130.6, 150.3

Magnesium Intake (%RDA)	128.2 (± 64.3)	119.1, 137.2


Among others, 23.3% of children were categorized as stunted, 57.5% with elevated BLLs, and 74.1% were living in the urban area (Table 2). The adequacy status of children’s nutrient intake was defined by intakes of energy, protein, zinc, vitamin A, vitamin C, vitamin D, calcium, phosphorus and magnesium. It was found that some intakes were inadequate for the children such as energy (62%), vitamin A (69%), vitamin C (85%), vitamin D (68%) and calcium (91%) (Table 3).

**Table 2:** Categorical distribution of the children’s characteristics (N = 193).


VARIABLE	N (%)

Stunting	

Not stunted	148 (76.7)

Stunted	45 (23.3)

Blood Lead Levels	

Normal (BLL < 5 µg/dL)	82 (42.5)

Elevated (BLL > 5 µg/dL)	111 (57.5)

Gender of child	

Girls	122 (63.2)

Boys	71 (36.8)

Child’s Age	

>5 years	174 (90.2)

<5 years	19 (9.8)

Childhood Diarrheal	

No chronic diarrheal history	188 (97.4)

Chronic diarrheal history	5 (2.6)

Mother’s Education	

Completed high school or higher	94 (48.7)

Completed secondary school or less	99 (51.3)

Mother’s age at birth	

Adult mother (20–34 years)	144 (69.4)

Young or advanced mother (< 20 years or > 35 years)	49 (30.6)

Child’s environment	

No environmental tobacco smoke	79 (40.9)

Environmental tobacco smoke	114 (59.1)

Residence	

Urban	143 (74.1)

Rural	50 (25.9)


**Table 3:** Categorical distribution of children’s nutrient intakes (N = 193).


VARIABLE	N (%)

Energy Intake	

Adequate (>80% RDA)	74 (38.3)

Inadequate (<80% RDA)	119 (61.7)

Protein intake	

Adequate (>80% RDA)	166 (86.0)

Inadequate (<80% RDA)	27 (14.0)

Zinc Intake	

Adequate (>80% RDA)	107 (55.4)

Inadequate (<80% RDA)	86 (44.6)

Vitamin A Intake	

Adequate (>80% RDA)	60 (31.1)

Inadequate (<80% RDA)	133 (68.9)

Vitamin C Intake	

Adequate (>80% RDA)	29 (15.0)

Inadequate (<80% RDA)	164 (85.0)

Vitamin D Intake	

Adequate (>80% RDA)	62 (32.1)

Inadequate (<80% RDA)	131 (67.9)

Calcium Intake	

Adequate (>80% RDA)	17 (8.8)

Inadequate (<80% RDA)	176 (91.2)

Phosphorus Intake	

Adequate (>80% RDA)	163 (84.5)

Inadequate (<80% RDA)	30 (15.5)

Magnesium Intake	

Adequate (>80% RDA)	145 (75.1)

Inadequate (<80% RDA)	48 (24.9)


Stunted status was found having association with children’s BLLs (p = <0.001) and with those living in rural residence (p = 0.008). However, stunted status has no association with gender (p = 1.00), children’s age (p = 0.78), history of chronic diarrheal (p = 0.33), mother’s education (p = 0.13), mother’s age at birth (p = 0.42) and exposure to tobacco smoke (p = 0.51) (Table 4).

**Table 4:** Association of Children’s Demographic Characteristics and Stunting Categories.


VARIABLE	NOT STUNTED, N (%)	STUNTED, N (%)	P-VALUE

Blood Lead Levels			

Normal (BLL < 5 µg/dL)	78 (52.7)	4 (8.9)	

Elevated (BLL > 5 µg/dL)	70 (47.3)	41 (91.1)	<0.001

Gender of child			

Girls	94 (63.5)	28 (62.2)	

Boys	54 (36.5)	17 (37.8)	1.00

Child’s Age			

>5 years	134 (90.5)	40 (88.9)	

<5 years	14 (9.5)	5 (11.1)	0.78

Child’s birth weight			

Normal birth weight	139 (93.9)	42 (93.3)	

Low birth weight	9 (6.1)	3 (6.7)	1.00

Childhood diarrheal			

No chronic diarrheal history	142 (98.0)	43 (95.6)	

History of chronic diarrheal	3 (2.0)	2 (4.4)	0.33

Mother’s Education			

Completed high school or higher	77 (52.0)	17 (37.8)	

Completed secondary school or less	71 (48.0)	28 (62.2)	0.13

Mother’s age at birth			

Adult mother (20–34 years)	113 (76.3)	31 (68.9)	

Young or advanced mother (< 20 years or > 35 years)	35 (23.7)	14 (31.1)	0.42

Child’s environment			

No environmental tobacco smoke	63 (42.6)	16 (35.6)	

Environmental tobacco smoke	85 (57.4)	29 (64.4)	0.51

Residence			

Urban	117 (79.0)	26 (57.8)	

Rural	31 (21.0)	19 (42.2)	0.008


The data revealed that the association of children’s stunted status and inadequacy among nutrient intakes was only found in vitamin A (p = <0.001) and phosphorus (p = 0.03) (Table 5).

**Table 5:** Association of Children’s Nutrient Intake (based on RDA) and Stunting Categories.


VARIABLE	NOT STUNTED, N (%)	STUNTED, N (%)	P-VALUE

Energy Intake			

Adequate (>80% RDA)	61 (41.2)	13 (28.9)	

Inadequate (<80% RDA)	87 (58.8)	32 (71.1)	0.19

Protein intake			

Adequate (>80% RDA)	131 (88.5)	35 (77.8)	

Inadequate (<80% RDA)	17 (11.5)	10 (22.2)	0.12

Zinc Intake			

Adequate (>80% RDA)	88 (59.5)	19 (42.2)	

Inadequate (<80% RDA)	60 (40.5)	26 (57.8)	0.06

Vitamin A Intake			

Adequate (>80% RDA)	56 (37.8)	4 (8.9)	

Inadequate (<80% RDA)	92 (62.2)	41 (91.1)	<0.001

Vitamin C Intake			

Adequate (>80% RDA)	23 (15.5)	6 (13.3)	

Inadequate (<80% RDA)	125 (84.5)	39 (86.7)	0.90

Vitamin D Intake			

Adequate (>80% RDA)	50 (33.8)	12 (26.7)	

Inadequate (<80% RDA)	98 (66.2)	33 (73.3)	0.48

Calcium Intake			

Adequate (>80% RDA)	16 (10.8)	1 (2.2)	

Inadequate (<80% RDA)	132 (89.2)	44 (97.8)	0.13

Phosphorus Intake			

Adequate (>80% RDA)	130 (87.8)	33 (73.3)	

Inadequate (<80% RDA)	18 (12.2)	12 (26.7)	0.03

Magnesium Intake			

Adequate (>80% RDA)	12 (75.7)	33 (73.3)	

Inadequate (<80% RDA)	36 (24.3)	12 (26.7)	0.90


Crude Odd Ratio (OR) and Adjusted OR analysis of children’s stunted status and BLLs and other predictors (Table 6). Elevated BLLs, rural residence, inadequate zinc, vitamin A, calcium and phosphorus intake were revealed to be substantially linked with stunting in bivariable logistic regression. Nevertheless, only increased BLL, rural inhabitants and inadequate vitamin A intake were substantially linked with stunting in multivariable analyses.

**Table 6:** Association between stunting and elevated BLL and Other Predictors.


VARIABLE	CRUDE OR	95% CI	P-VALUE	ADJUSTED OR	95% CI	P-VALUE

Blood Lead Levels						

Elevated (BLL > 5 µg/dL)	11.4	3.9–33.5	<0.001	9.8	3.1–30.7	<0.001

Mother’s Education						

Completed junior secondary school or less	1.8	0.9–3.5	0.10	1.0	0.4–2.2	0.96

Residence						

Rural	2.8	1.4–5.6	0.005	3.7	1.6–8.9	0.003

Energy Intake						

<80% RDA	1.7	0.8–3.6	0.14	2.2	0.8–5.7	0.12

Protein intake						

<80% RDA	2.2	0.9–5.3	0.07	0.8	0.2–2.9	0.71

Zinc Intake						

<80% RDA	2.0	1.0–3.9	0.04	0.6	0.2–1.7	0.39

Vitamin A Intake						

<80% RDA	6.2	2.1–18.4	0.001	5.4	1.4–20.3	0.01

Calcium Intake						

<80% RDA	2.6	1.2–6.0	0.02	1.3	0.1–15.0	0.83

Phosphorus Intake						

<80% RDA	2.6	1.2–6.0	0.02	1.8	0.5–6.0	0.37


Bivariate analysis results indicate a significant relationship between increased BLLs and stunting [OR: 11.4 (95% CI: 3.9–33.5); p = 0.001]. Also, the multiple logistic regression models reveal that higher BLLs was a statistically significant and independent predictor of stunting after controlling for mother’s education, residency, calories, protein, zinc, vitamin A, calcium and phosphorus intake. When compared to those with normal BLLs, the odds of being stunted were approximately 10 times higher [AOR = 9.8 (95% CI: 3.1–30.7); p = 0.001]. Compared to urban inhabitants, those who lived in rural areas had the odds of stunting 3.7 times (95% CI: 1.6–8.9), p = 0.003. When compared to children whose vitamin A levels were adequate, children with inadequate vitamin A had 5.4 times the risk of being stunted (95% CI: 1.4–20.3), p = 0.01. No interaction between a mother’s education and her children’s nutrient intake was found in the interaction assessment (p > 0.05).

## Discussion

The findings of this study show a link between stunting at ages two to nine years and elevated BLLs. The BLLs were found to be an independent predictor of stunting (p = 0.0001). This result was consistent with the tendency that children’s exposure to harmful environments over time causes a steady accumulation of lead, which results in chronic stunting. Several earlier research indicated the connection between lead exposure in children and stunting, which is like the findings of the current study [8,15,16]. Our findings also demonstrate that the association between high BLLs and stunting persisted even after we considered the potential confounding effects of several variables, such as the mother’s level of education andplace of residence, as well as energy, protein, zinc, vitamin A, calcium and phosphorus intake. This result demonstrated that BLLs were not associated with stature through collinearity with other demographic and dietary factors. Other research has produced the same conclusion, suggesting that lead’s direct impact on a child’s growth may be the cause of the link between BLLs and stunting. In contrast to its collinearity with other demographic and dietary variables, BLLs is linked with stature [14]. Although sociodemographic [17] and maternal factors [16] were significant confounders in prior studies that examined the connection between stunting and BLL, they were not in the current study. Nonetheless, our results concurred with those of a different study conducted in Bangladesh slum areas [7], and stunting could be linked to lead’s direct impact on a child’s growth. The average BLLs of children in this study were lower than the average BLLs of children living near recycled lead-acid battery smelters in the District of Tegal, Central Java. However, when compared to earlier studies conducted in Indonesia (5.5 µg/dL vs. 39.3 µg/dL, respectively) [18], the proportion of kids with BLLs under 5 µg/dL was higher than that of kids living in Jakarta’s recycling of spent lead-acid batteries (57.5% vs. 47.3%, respectively) [19].

When compared to data from the 2018 Indonesian Basic Health Research Report, the proportion of stunting in our study locations (two of West Bangka’s subdistricts) was greater (26.3% ; 23.4%, respectively) for age under five years, and 23.0% ; 18.7%, respectively, for age group 5–12 years) [20]. Child growth status is a reliable surrogate indicator of community health status including levels of environmental pollution [21] and stunting is a form of malnutrition especially related to protein and energy deficiency [22], which is also supported by other studies that it has an association with vitamin deficiency. Most children who suffer from stunting are deficient in many micronutrients with mostly deficient in vitamin A [23,24,25]. Protein and energy, according to our data, were not substantially related to stunting (p = 0.12 and p = 0.19, respectively). We discovered that the correlation between stunting and vitamin A deficiency was statistically significant (p = 0.014) among the several micronutrient deficiencies that were identified as possibly associated with stunting. In this study population, dietary intakes of vitamin A were generally low. The average consumption was 81 mg compared to the RDA of 400–500 mg (varying for ages 2–9 years), and 68% of children had vitamin A deficiencies because their intake fell below the RDA by more than 80%.

Vitamin A insufficiency results from insufficient consumption of the vitamin to meet physiological requirements. Even a minor subclinical vitamin A deficiency might cause health issues. It may impede bone development, slow growth rates, and reduce the likelihood of surviving serious illness [26]. In the children sampled who were lacking, it might have increased the likelihood of stunting. Growth retardation is linked to reduced vitamin A consumption because it lowers IGF-1 serum production, which triggers the release of nocturnal growth hormone [27]. Retinoic acid, the active metabolite of vitamin A, is a growth hormone gene regulator and is essential for growth hormone secretion; as a result, a vitamin A deficiency results in impaired growth hormone synthesis and secretion in the pituitary growth hormone cells, which causes somatic growth failure, especially in preschoolers. In relation with stunting, we did not conduct a test to prove the possibility of an interaction between BLL and vitamin A because it was not supported by adequate theory. Moreover, children from rural areas are more susceptible to stunting. According to our study, stunting was also related to where kids lived (p = 0.003). According to a literature study, residing in rural areas and having limited access to healthcare are linked to child stunting in Indonesia [28]. In previous studies it was also reported that stunting is more prone to occur in children in rural Indonesia which is related to poverty and inadequacy in disease management [29]. Muntok, the capital of the West Bangka District, might have better health care infrastructure than Simpang Teritip. The accessibility of medical supplies, water and sanitation facilities, and other social and societal factors linked to child stunting, however, were not evaluated. In addition, the mean BLL (±SD) children in rural areas was higher than in urban areas (6.3 ± 3.2 µg/dL vs 5.2 ± 2.3 µg/dL). In contrast, the findings of a previous study conducted in Bangladesh revealed that children’s mean BLLs in urban areas were greater than those in rural areas (13.5 ± 8.2 µg/dL vs 7.3 ± 6.3 µg/dL) [17]. The various study sites might be to blame for this discrepancy. According to a study conducted in Bangladesh, lead poisoning was more common among urban children than rural children, presumably because of the industries’ quick economic development and environmental degradation. The tin mining exposure most likely contributed to the higher BLL seen in study participants. Mining operations may contaminate the air, water, and soil with lead.

## Limitation

During mining operations, and after the mine was closed, lead contaminants emitted from it had an impact on the surrounding environment. Even though our research focused on children’s homes and blood lead levels, the levels of lead in polluted environments remained unknown. For evaluating the environmental impact of pollution, the levels of heavy metals in contaminated soil, water and air were crucial. Also, depending on the climate, geography and elevation of the site for soils, different locations have different levels of heavy metal pollution. Our inability to fully describe the ambient conditions at the study site limits the scope of our investigation.

## Conclusion

Between the ages of two and nine years, there is a strong correlation between stunting and blood lead levels. When accounting for the mother’s level of education, place of residence and dietary consumption of energy, protein, zinc, vitamin A, calcium, and phosphorus, this link is marginally attenuated. The research suggested that raising children near tin mining is harmful to their growth.
